# Radiological lung sequelae, functional status and symptoms in older patients 3 and 6 months after hospitalization for COVID-19 pneumonia

**DOI:** 10.1007/s11739-023-03259-y

**Published:** 2023-04-06

**Authors:** Chiara Di Pentima, Sara Cecchini, Francesco Spannella, Federico Giulietti, Massimiliano Allevi, Paola Schiavi, Francesca Carnevali, Lorenzo Zoppi, Maria Carmela Ciociola, Fiammetta Ventura, Gina Dragano, Piero Giordano, Enrico Paci, Riccardo Sarzani

**Affiliations:** 1 Internal Medicine and Geriatrics, IRCCS INRCA, via Della Montagnola n. 81, 60127 Ancona, Italy; 2 Department of Clinical and Molecular Sciences, University “Politecnica Delle Marche”, via Tronto 10/a, Ancona, Italy; 3 Department of Radiology, IRCCS INRCA, via Della Montagnola 81, Ancona, Italy

**Keywords:** COVID-19, SARS-COV-2, Older adults, Chest CT, Functional status, Disability

## Abstract

The aim of our study was to assess the lung sequelae and clinical consequences 3 and 6 months after hospitalization for COVID-19 pneumonia in older patients. An observational study was conducted on 55 patients aged 65 years and older. Activities of daily living (ADL) and clinical frailty scale (CFS) were assessed at baseline and after 3 months. Both quantitative assessment at chest high-resolution computed tomography (CT) and semi-quantitative severity score (CTSS) were performed at baseline and after 3 and 6 months. Mean age: 82.3 ± 7.1 years. Male prevalence: 56.4%. After 6 months, ground-glass opacities (GGO) were still detectable in 22% of subjects, while consolidations were no longer appreciable. During follow-up, CTSS reached an overall median score of zero after 6 months. Fibrotic-like changes were found in 40% of subjects with an overall median score of 0 (0–5) points, being more prevalent in males. Patients reporting worsening ADL and CFS were 10.9% and 45.5%, respectively. They were associated with the burden of comorbidities, especially history of heart failure and chronic obstructive pulmonary disease at baseline. Amnesic disorders, exertional dyspnea, and fatigue were the most relevant symptoms reported. No association emerged between persistent or new-onset symptoms and evidence of fibrotic-like changes. The typical chest CT abnormalities of the COVID-19 pneumonia acute phase resolved in most of our older patients. Mild fibrotic-like changes persisted in less than half of the patients, especially males, without significantly affecting the functional status and frailty condition, which instead were more likely associated with pre-existing comorbidities.

**Supplementary Information:**

The online version contains supplementary material available at 10.1007/s11739-023-03259-y.

## Introduction

The global pandemic caused by the severe acute respiratory syndrome coronavirus 2 (SARS-CoV-2) has affected millions of people and caused millions of deaths worldwide. Since the beginning of the pandemic, older age, besides the presence of comorbidities, has been clearly associated with a worse outcome in coronavirus disease 2019 (COVID-19) [1, 2]. Pathogenesis, clinical characteristics, and complications of SARS-CoV-2 pneumonia have been widely described [3–5], but the long-term COVID-19 consequences are still uncertain, especially in the older population. According to the World Health Organization (WHO), the COVID-19 recovery process may lead to a “post-COVID” syndrome, as a symptom set lasting more than 2 months from disease onset, not referable to alternative diagnoses. Among post-COVID-19 sequelae, fatigue, exertional dyspnea, chest pain, cough, anxious and depressive state, amnesic disorders, and attention deficits have been described as the main symptoms [6–8]. A reduction in the activities of daily living (ADL) was also reported [9].

Chest high-resolution computed tomography (HRCT) is the reasonable tool for initial diagnosis of COVID-19 pneumonia and estimation of disease severity [10]. The predominant CT findings are bilateral, peripheral, and basal predominant ground-glass opacities (GGO), consolidations, or both [11]. Several previous studies had discussed extensively the radiological features of the disease at different stages [12, 13], but few studies focused on radiological sequelae during recovery and follow-up. Radiological sequelae, such as residual GGO or fibrotic-like abnormalities, need to be investigated, especially in older patients with comorbidities and at increased risk of developing extensive disease and critical illness. However, most of the studies on these aspects focused on younger/adult population [14–16].

Although possible COVID-19 follow-up program modalities have been indicated by multiple scientific societies and consensus [7, 17] and several observational follow-up studies have been performed so far [18–23], they are still heterogeneous and none of these focused on older patients. Moreover, no previous studies have performed a detailed quantification and description of lung CT findings to evaluate their extension and evolution over time in association with symptoms and burden of comorbidities in older patients. Therefore, we aimed at describing clinical, functional and radiological consequences of COVID-19 3 and 6 months after hospitalization for SARS-COV-2-related pneumonia in older adults.

## Methods

### Study design and participants

We performed a longitudinal observational study on 55 older adults consecutively hospitalized, between January and May 2021, in the COVID-19 wards of the “Istituto di Ricovero e Cura a Carattere Scientifico Istituto Nazionale di Riposo e Cura per Anziani” (IRCCS INRCA), Ancona, (Italy), for SARS-CoV-2-related pneumonia. Our institute is the only organization specifically focused on geriatric care and gerontological research in Italy. Our hospital is dedicated to scientific research and care of older subjects (mostly aged 80 years and older). We took into account the following inclusion criteria: age ≥ 65 years, hospitalization for COVID-19 pneumonia, chest HRCT performed during hospitalization, survivors of acute disease, and ability to perform follow-up assessment. The diagnosis of SARS-COV-2-related pneumonia was based on the virus detection by reverse transcriptase-polymerase chain reaction (RT-PCR) from a nasal/oro-pharyngeal swab on admission and the presence of at least one new radiological finding of pneumonia (i.e., ground-glass opacities, crazy-paving pattern, lobular and sub-segmental areas of consolidation) on chest X-ray or chest HRCT during hospitalization. We excluded patients unable to be followed due to existing medical conditions, limited access to the health service or terminally ill patients (end-stage renal disease or dialysis, decompensated cirrhosis, advanced cancer, severe dementia, bed-rest syndrome). Eligible patients were re-evaluated 3 and 6 months after discharge. In particular, functional status, frailty condition and symptoms were re-evaluated after 3 months, while chest HRCT was performed 3 and 6 months after discharge.

### Clinical parameters

Clinical and laboratory parameters, chest HRCT findings and treatments during hospitalization were collected from electronic clinical records. The following anamnestic data were taken into account: history of ischemic heart disease, arterial hypertension, history of heart failure (HF), chronic obstructive pulmonary disease (COPD), peripheral artery disease, stroke/transient ischemic attack (TIA), type 2 diabetes mellitus, and cognitive impairment. To evaluate the burden of comorbidities, both Charlson Comorbidity Index (CCI) [24] and Geriatric Index of Comorbidity (GIC) (classes I–II were categorized as “low comorbidity”, classes III–IV as “high comorbidity”) [25] were performed at baseline. Polypharmacy was defined as the use of five or more drugs before hospital admission. Functional status was evaluated with the activities of daily living (ADL) hierarchy scale referring to the 2 weeks before hospital admission (scores of 1–2 stood for functional dependency, 3–4 for partial functional dependency, 5–6 for independency) [26], while frailty condition was evaluated with the clinical frailty scale (CFS) [27]. Both ADL and CFS, together with symptoms, were evaluated at baseline (during hospitalization) and 3 months after discharge. Persistent or new-onset symptoms were discriminated from comorbidities-related pre-existent symptoms and their presence/absence was considered based on answering to specific questions. We took into account the following symptoms: fatigue, arthralgia, exertional dyspnea, resting dyspnea, chest pain, palpitations, depressive mood, anxiety, and amnesic disorders.

### Radiological analysis

Volumetric chest HRCT examinations were performed in the supine position at full inspiration without contrast agent. Baseline CT scans are usually performed within 48 h from hospital admission on a 16-slice MDCT scanner (GE BrightSpeed Elite). Scanning parameters were 120 kV and 100–200 mA with a slice thickness of 1.0 mm and a matrix size of 512 × 512 pixels. Images were reconstructed with a sharp reconstruction kernel for parenchyma. The lung window setting was at a window level of 600 Hounsfield units (HU) and window width of 1600 HU. Three- and 6-month follow-up scans were obtained by a 16-slice MDCT scanner with the following parameters: kvp: 140 kvp, mAs: 170, gantry rotation time: 0.5 secs, and slice thickness: 1 mm.

#### Qualitative and semi-quantitative assessment

All chest CT findings were defined according to the Fleischner Society glossary and the qualitative evaluation included the presence of the following CT pulmonary abnormalities: ground-glass opacities (GGO), consolidations, interlobular thickenings, linear atelectasis, pleural parenchymal band, bronchiectasis/bronchiolectasis, reticulation, pulmonary nodules, pleural and pericardial effusion, and lymphadenopathies [28]. The CT evidence of fibrotic-like changes was defined as the presence of subpleural parenchymal bands, linear atelectasis, bronchiectasis/bronchiolectasis, and/or honeycombing [15]. Structural alterations have been considered as post-infection CT abnormalities if confined to the same location as the initial CT findings of the affected lung areas during acute disease.

To quantify the extent of pulmonary abnormalities and fibrotic-like changes, we used the semi-quantitative CT severity score (CTSS) based on the degree of lung lobe involvement (0: 0%; 1: < 5%; 2: 5–25%; 3: 26–49%; 4: 50–75%; 5: > 75%; range 0–5 for each lobe; global score range 0–25) [29]. Based on CTSS, the pulmonary involvement was categorized into three groups: mild (0–7), moderate (8–15), and severe (15–25).

#### Quantitative assessment

Primary image data sets were transferred to the PACS workstation and evaluated using thoracic VCAR software (GE Healthcare, Chicago, IL, USA). Thoracic VCAR software is a CE-marked medical device originally designed to quantify pulmonary emphysema in patients with chronic obstructive pulmonary disease and recently adapted for use in chest CT affected by COVID-19 infections in clinical practice. The software provides quantification of the emphysema, healthy residual lung parenchyma, GGO, and consolidation based on Hounsfield unit. It also provides segmentation of the lungs and of the airway tree and calculation of the total volumes for both the right and left lung [30]. Lung parenchyma was divided by HU intervals: from − 1024 to less than − 977 HU, representing emphysematous changes; from − 977 to − 703 HU, representing normal parenchyma; from − 703 to − 368 HU, representing ground-glass opacity; values higher than − 100 to 5 HU, representing consolidations; the remaining lung parenchyma is classified as other. The software automatically calculates the healthy lung volume, the ground-glass opacities and the consolidations, which were expressed both in liters and as percentage.

### Statistical analysis

Data were analyzed using the Statistical Package for Social Science version 21 (SPSS Inc., Chicago, Illinois, USA). A value of *p* < 0.05 was defined as statistically significant. Continuous variables were checked for normality and expressed as mean ± standard deviation or median and interquartile range for significantly skewed variables. Categorical variables were expressed as percentages. The *χ*^2^ test was used to analyze the differences between categorical variables. T student or analysis of variance (ANOVA) test and Mann–Whitney or Kruskal–Wallis test were used to compare continuous variables with normal and non-normal distribution, respectively. Mc Neman test, Friedman test, and the two-sided test marginal homogeneity were used to assess the differences of the selected variables at the specified time intervals.

## Results

### General baseline characteristics on admission

The general baseline characteristics of the study population are summarized in Table 1. The mean age was 82.3 ± 7.1 years, with male prevalence (56.4%). Median days from symptom onset to hospital admission were 7 (4–10) and the mean length of stay was 19.0 ± 6.9 days. The more prevalent comorbidities were arterial hypertension, dyslipidemia, anemia, and type 2 diabetes mellitus. Polypharmacy was found in almost half of the patients (49.1%). Most patients were independent in ADL (ADL 5–6) before hospitalization. All patients (100%) took corticosteroids during hospitalization (dexamethasone 6 mg once daily) and 54.5% of patients took antiviral therapy (remdesivir).

**Table 1:** General baseline characteristics of the study population

General baseline characteristics	N° 55 patients
Age (years)	82.3 ± 7.1
Sex (male)	56.4%
Overweight/obesity (BMI > 25 kg/mq)	59.6%
Active/former smoking	27.3%
CCI	6 (4–7)
GIC classes I–II	90.9%
GIC classes III–IV	9.1%
CFS	3.6 ± 1.3
ADL 5–6	78.2%
ADL 3–4	20.0%
ADL 1–2	1.8%
Main comorbidities	
Arterial hypertension	78.2%
Dyslipidemia	45.5%
Anemia (Hb < 12 g/dl)	38.2%
Type 2 diabetes mellitus	36.4%
Ischemic heart disease	23.6%
Peripheral artery disease	18.2%
History of heart failure	16.4%
Cognitive impairment	16.4%
COPD	14.5%
Atrial fibrillation	14.5%
Previous stroke/TIA	9.1%
Admission laboratory parameters	
Hb (g/dl)	12.7 ± 1.7
WBC (n/microl)	7290 ± 3332
PLT (n/microl)	214,000 ± 88,535
Neutrophils (n/microl)	5548 ± 3073
Lymphocytes (n/microl)	700 (550–940)
eGFR (ml/min/1.73m^2^)	69.6 ± 28.0
Ferritin (mcg/l)	566 (298–1095)
CRP (mg/dl)	9.9 ± 5.5
Hs-troponin T (pg/ml)	22 (13–34)
NT-proBNP (pg/ml)	772 (395–1884)
D-dimer (mcg/l)	1275 (695–2680)
Admission arterial blood gas parameters	
pH	7.47 ± 0.44
pO2 (mmHg)	65.4 ± 12.2
pCO2 (mmHg)	34.0 ± 4.6
HCO3- (mmol/l)	25.3 ± 4.1
sO2 (%)	94.4 ± 3.7
FIO2 (%)	21 (21–32)
Lactate (mmol/l)	1.1 (0.8–1.3)
P/F (mmHg)	262 ± 85
Worst P/F (mmHg) during hospitalization	177 ± 66
Respiratory support needed during hospitalization	
Oxygen therapy	54.5%
HFNC/CPAP	21.8%
NIV	23.6%
In-hospital treatment for COVID-19	
Azithromycin	63.6%
Remdesivir	54.5%
Corticosteroids	100.0%
Anticoagulants at prophylactic dose	52.7%
Anticoagulants at therapeutic dose	45.5%

*CCI* Charlson Comorbidity Index, *GIC* Geriatric Index of Comorbidity, *CFS* clinical frailty scale, *ADL* activities of daily living, *Hb* hemoglobin, *COPD* chronic obstructive pulmonary disease, *TIA* transient ischemic attack, *WBC* white blood cells count, *PLT* platelets count, *eGFR* estimated glomerular filtration rate, *CRP* C-reactive protein, *NT-proBNP* N-terminal pro-B-type natriuretic peptide, *P/F* ratio of arterial oxygen partial pressure (PaO2 in mmHg) to fractional inspired oxygen (FiO2 expressed as a fraction, not a percentage), *HFNC/CPAP* high flow nasal cannula/continuous positive airway pressure, *NIV* non-invasive ventilation

The baseline characteristics of the study population according to clinical severity, expressed through the respiratory support needed during hospitalization [oxygen therapy (54.6%), high flow nasal cannula/continuous positive airway pressure (HFNC/CPAP) (21.8%), and non-invasive ventilation (NIV) (23.6%)], are described in Supplemental Table S1. Patients with greater clinical severity had higher C-reactive protein (CRP) levels, worse PaO_2_/FiO_2_ ratio (the ratio of arterial oxygen partial pressure in mmHg to fractional inspired oxygen), and longer length of stay [23.1 ± 8.0 days for subjects treated with NIV, 23.1 ± 5.2 days for subjects treated with HFNC/CPAP, and 15.8 ± 5.1 days for subjects treated with oxygen therapy, respectively (*p* < 0.001)]. Patients with greater clinical severity had also higher prevalence of treatment with azithromycin during hospitalization (Supplemental Table S1).

On admission, 32.1% of patients had mild lung involvement at CTSS of pulmonary abnormalities, 54.5% had a moderate involvement, and 12.7% had a severe involvement. The most common radiological patterns on admission were GGO (100% of patients) and crazy paving (90% of patients), followed by consolidations (49% of patients) and mixed pattern (47.3% of patients). The baseline characteristics of the study population according to admission lung involvement at CTSS are described in Supplemental Table S2. Higher CTSS of pulmonary abnormalities were related to higher CRP levels. Time from symptom onset to baseline CT on admission was significantly different between mild, moderate, and severe CTSS of pulmonary abnormalities: the severity increased as the days from symptom onset increased [6 (3–8) days, 7 (6–11) days and 9 (3–12) days, respectively (*p* = 0.008)].

### Radiological findings at follow-up

#### Qualitative and semi-quantitative analyses

**Fig. 1:**
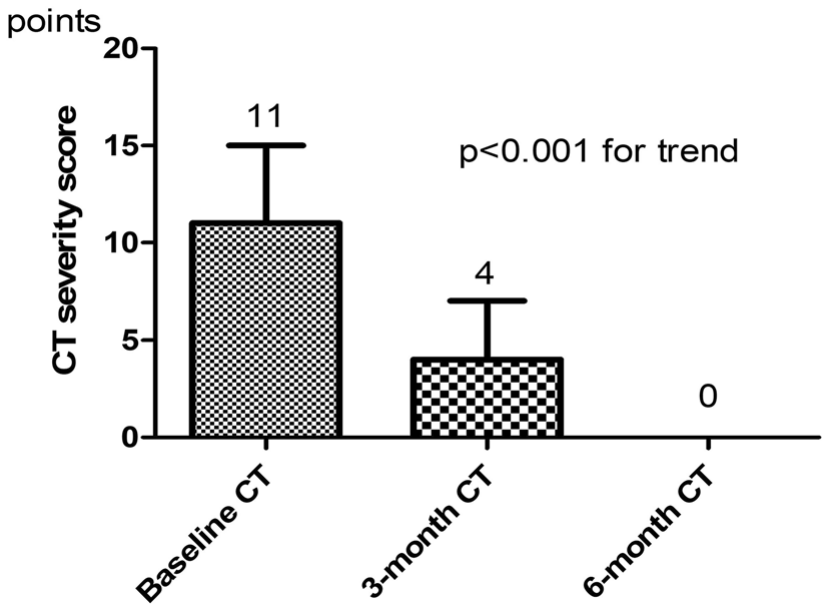
Trend of semi-quantitative CT severity score at follow-up (median and interquartile range)

At the 3-month follow-up, the prevalence of diffuse GGO was still high; conversely, the frequency of consolidations was low and none of the subjects showed a crazy-paving pattern (Table 2). At the 6-month follow-up, GGO was still detectable in 22% of the subjects, while consolidations were no longer appreciable. At the 3-month follow-up, 13% of patients were categorized as severe at CTSS, 55% as moderate, and 33% as mild. At the 6-month follow-up, 16.7% of patients had moderate CTSS and 83.3% had mild CTSS, while no patients had severe CTSS. A significant reduction in CTSS, as absolute value, was observed during follow-up CT scans, reaching an overall median score of 0 after 6 months (Fig. 1).

**Table 2:** Trends of lung CT abnormalities and semi-quantitative CT severity score at follow-up

CT finding	Baseline CT	3-month CT	6-month CT
GGO	100%	92%^†^	22%^†^
Consolidations	49%	14%*	0%^†^
Crazy paving	90%	0%^†^	0%^†^
Reticulation	16%	76%*	66%*
Bronchiectasis	11%	48%*	29%*
Pleural effusion	16%	14%	0%^†^
Fibrotic-like changes (parenchymal bands, traction bronchiectasis, and/or honeycombing)	5%	65%*	40%*

*GGO* ground-glass opacity

*p < 0.05 (comparison referred to baseline)

^†^Statistical analysis not applicable

Evidence of fibrotic-like changes was found on follow-up CT scans in 22 of the 55 participants (40%). Of these, 13 (59%) subjects had mild and 9 (41%) subjects had moderate CTSS for fibrotic-like abnormalities, with an overall median score for fibrotic-like changes of 0 (0–5) points after 6 months. None of the subjects presented severe fibrotic lung disease. Regarding clinical and laboratory parameters, no association emerged with the presence of fibrotic-like changes after 6 months, except for sex. Indeed, males had a higher probability of fibrotic-like changes than females (OR: 3.2, 95% CI 1.0–10.2, *p* = 0.046). No association emerged between fibrotic-like changes at follow-up and in-hospital treatments for COVID-19 (Supplemental Table S3).

#### Quantitative analysis

Data from CT quantitative analysis performed at baseline, and after 3 and 6 months, expressed as percentage of lung parenchyma volume with abnormalities on the total lung volume are described in Table 3. During the follow-up, the percentage of lung parenchyma affected by GGO and consolidations dropped significantly. In particular, the baseline median GGO and consolidations extension decreased from 32.2% to 9.8% and from 5.3% to 1.2%, respectively, after 6 months, with well-aerated lung volume reaching almost 90% in median of the total lung volume.

**Table 3:** Trends of pulmonary abnormalities at CT quantitative analysis during follow-up

CT pulmonary volumes	Baseline CT	3-month CT	6-month CT	*p*
Normal parenchyma (%)	60.1 (40.7–70.0)	80.1 (70.5–86.2)	87.2 (80.6–89.8)	< 0.001
GGO (%)	32.2 (24.3–46.1)	15.6 (11.1–23.0)	9.8 (7.6–15.6)	< 0.001
Consolidations (%)	5.3 (3.4–11.0)	2.4 (1.5–3.3)	1.2 (0.9–2.7)	< 0.001

*GGO* ground-glass opacity

### Clinical parameters at follow-up

**Fig. 2:**
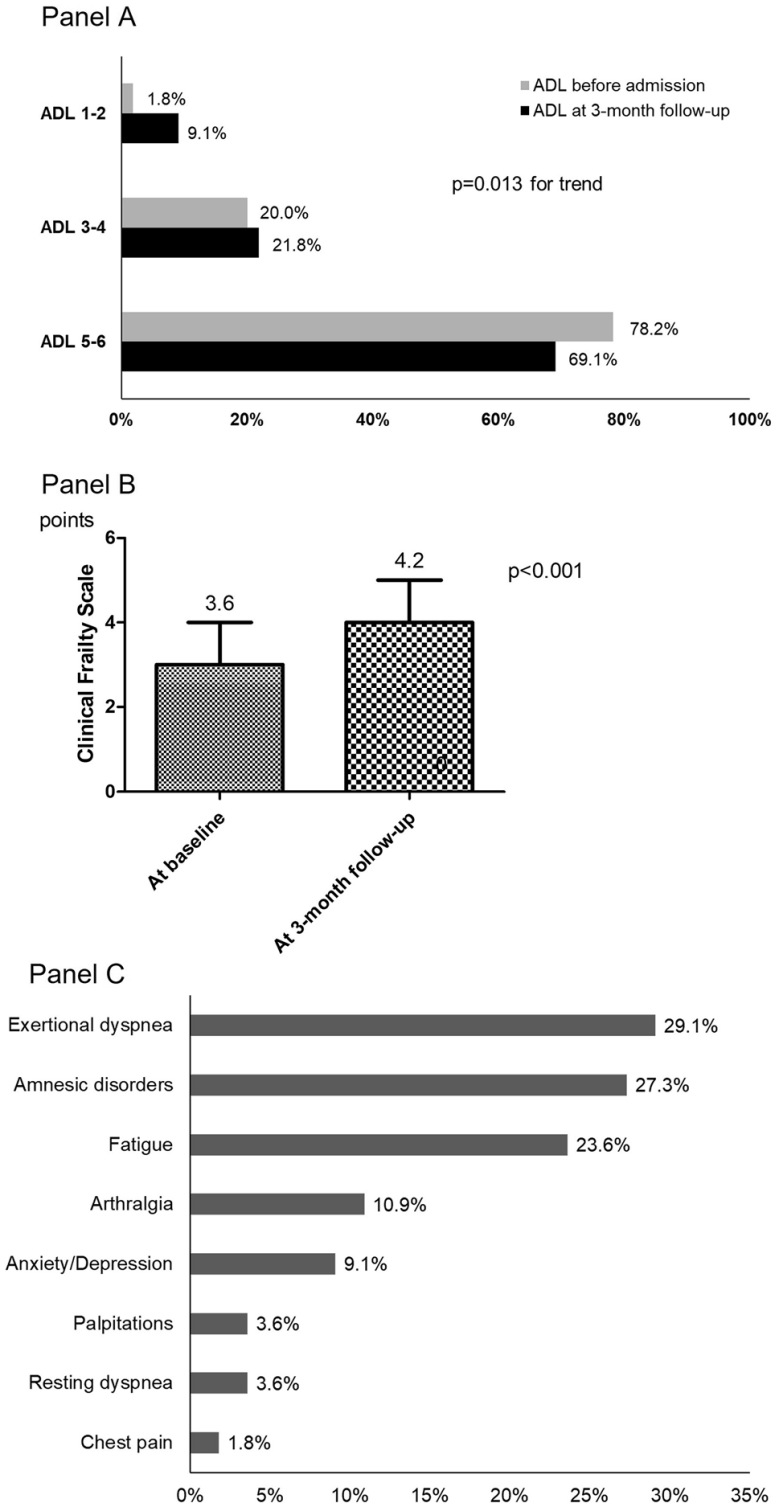
Clinical parameters at follow-up; **A** trend of functional status evaluated with activities of daily living (ADL); **B** trend of clinical frailty scale (mean standard ± deviation); **C** prevalence of persistent or new-onset symptoms at follow-up

At the 3-month follow-up, 10.9% of patients reported a worsening functional status compared to baseline, with an increase in functional dependency (Fig. 2A). At the same time, mean CFS increased (Fig. 2B) with 45.5% of subjects showing a worsening of frailty condition. Regarding persistent or new-onset symptoms, amnesic disorders, exertional dyspnea, and fatigue were the most relevant (Fig. 2C). Less than half of the patients (41.8%) reported no residual or new-onset symptoms.

General baseline characteristics of the study population according to the trend of functional status and frailty condition are described in Supplemental Table S4. Patients with increased functional disability had higher pre-admission CCI and CFS, higher prevalence of smoking, HF, and COPD as comorbidities. Patients with a worsening of functional status and frailty condition had higher prevalence of treatment with anticoagulants at therapeutic dose during hospitalization. Patients with a longer hospital stay tended to have worsening of frailty condition at follow-up (17.5 ± 6.3 days vs 21.0 ± 7.2 days, *p* = 0.060).

Regarding the association between changes in functional/frailty status and persistent or new-onset symptoms, patients with fatigue or anxiety/depression had higher probability of having an increase in functional disability (30.8% vs 4.8%, *p* = 0.023 for fatigue and 40.0% vs 8.0%, *p* = 0.029 for anxiety/depression) and a worsening of frailty condition (31.0% vs 92.3%, *p* < 0.001 for fatigue and 100.0% vs 40.0%, *p* = 0.015 for anxiety/depression). An association between the presence of exertional dyspnea and worsening CFS was also found (56.0% vs 44.0%, *p* < 0.001). No association emerged between persistent or new-onset symptoms, evidence of fibrotic-like changes, and changes in functional or frailty status (all *p* > 0.05).

## Discussion

**Fig. 3:**
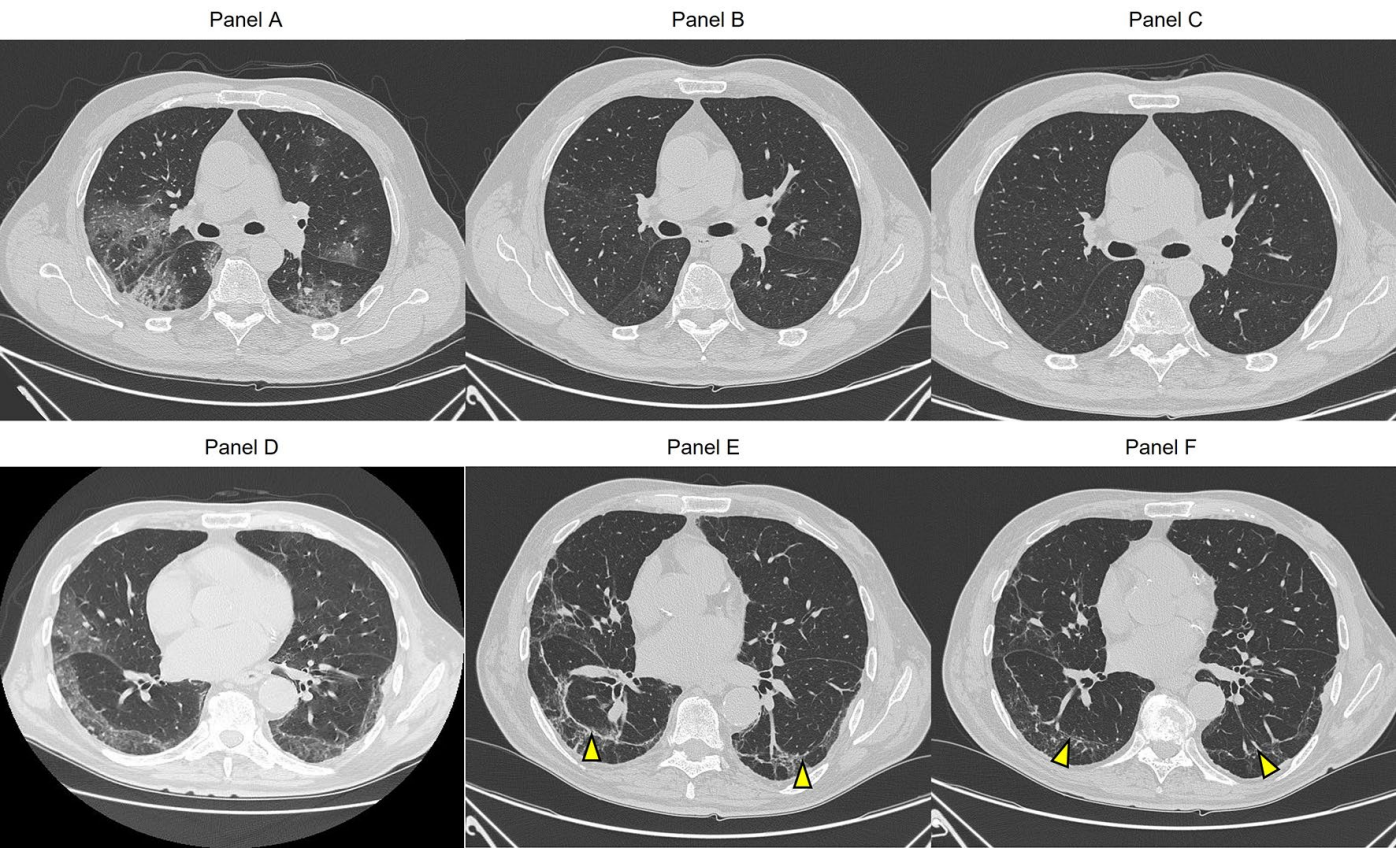
High-resolution chest CT findings in a subject with complete resolution (top) and in a subject with persistent pulmonary abnormalities (bottom) at follow-up; **A** axial section at baseline with diffuse bilateral hazy GGOs in the posterior peripheral subpleural region; **B** residual disease as an area of mild GGOs at the 3-month follow-up in the superior segment of right lower lobe; **C** complete resolution of the pulmonary lesions at the 6-month follow-up; **D** scans obtained at baseline with multiple GGOs and interstitial thickening involving the middle lobe and lower lobe of the right and left lung; **E** scans obtained at the 3-month follow-up with partial absorption of the GGOs and appearance of traction bronchiectasis and parenchymal bands; **F** scan obtained at the 6-month follow-up with the persistence of thinning parenchymal bands and thickening of the adjacent pleura (arrows)

We evaluated the clinical and radiological consequences of SARS-COV-2 infection in older patients hospitalized for COVID-19 pneumonia and their impact on functional status and frailty during a follow-up of 6 months. To the best of our knowledge, this is the first study that evaluated the clinical sequelae of COVID-19 pneumonia in such older population and with such detailed radiological characterization, which allowed us to evaluate the true extent of the residual lung lesions. These aspects were investigated in an older comorbid population with high vulnerability, in which scientific evidence is still scarce. In our sample, evidence of fibrotic-like changes was observed in 22 of the 55 participants (40%), whereas residual GGO remained in 12 of 55 participants (22%). However, in patients with persistent GGO at 6-month follow-up, quantitative analysis revealed minimal CT abnormalities reflected by a total CTSS ≤ 5 and a marked decrease in GGO density, named as the “tinted” sign or “melted sugar sign” (Fig. 3). According to previous studies on the long-term pulmonary consequences of COVID-19, imaging signs of improvement at follow-up CT included reduction in the number and size or resolution of GGOs and a decreased attenuation of GGO (melted sugar sign), which may indicate the gradual regression of the inflammation infiltrates, edema, or hemorrhaging and re-expansion of the alveoli [31]. This favorable evolution of the radiological features, with only minor parts of the lung parenchyma still involved by alterations at follow-up, was also found in our older population. Meta-analyses of studies on younger populations showed pooled prevalence of CT abnormalities ranging from 40 to 70% [14, 16]. GGOs were the most frequent CT abnormalities and a wide variability was present among the studies depending on the population taken into account and follow-up timing [14, 16].

In a prospective cohort study on 114 patients with severe SARS-CoV-2 pneumonia, Han et al. reported residual CT abnormalities in 62% of participants after 6 months, 35% of which were “fibrotic-like” features (parenchymal bands, traction bronchiectasis, and/or honeycombing) and 27% residual GGO. They found that fibrotic-like changes were associated with older age, acute respiratory distress syndrome, longer hospital stays, tachycardia, non-invasive mechanical ventilation, and higher initial chest CT score [15]. Another study on patients with COVID-19 pneumonia highlighted the minor role of aging as risk factor for fibrotic-like changes compared to the more important role played by pneumonia severity (need for ventilation and less extensive well-aerated lung during acute disease) [32]. However, no association emerged between fibrotic-like changes and clinical or laboratory parameters in our analysis. Despite the older age of our population, the prevalence of fibrotic-like changes was lower than the aforementioned studies. This could be explained by the low rate of severe involvement at baseline CT (12.7%) in our sample, likely due to the high intra-hospital mortality rate which prevented the inclusion in this longitudinal study [33]. The CovILD Study [34] analyzed 1-year CT abnormalities after COVID-19 pneumonia in a younger population (mean age: 59 ± 13 years). The authors found that most pulmonary alterations regressed, but a substantial number of patients (59%) showed persistent CT abnormalities after 1 year, that were predominantly represented by only subtle subpleural reticulation. In line with the CovILD study [34], our radiological quantitative analysis well documented that abnormalities involved less than 10% of total lung volume after 6 months. Therefore, nearly all participants with CT abnormalities had a very limited disease. Fibrotic-like changes were localized regionally with areas of prior GGOs and were mainly represented by subpleural parenchymal bands and bronchiectasis with no evidence of honeycombing (Fig. 3). Notably, none of the participants presented severe fibrotic lung disease. Other series of patients with moderate COVID-19 pneumonia showed no evidence of fibrosis at 1-year chest CT [35]. Our study is a useful addition to the evidence-based literature on this topic in older patients, although major uncertainty remains on the clinical significance of the medium- to long-term residual abnormalities at chest CT after acute COVID-19 pneumonia. In our older population, no association emerged between fibrotic-like changes and persistent or new-onset symptoms or changes in functional or frailty status.

While on the one hand most patients showed an improvement or almost a complete resolution of radiological pneumonia signs, on the other hand a significant deterioration of the functional status, especially of the frailty condition, was found in our older population. Similarly to other studies on both younger and older populations [22, 36–41], neurological disorders, dyspnea, and fatigue were the most relevant symptoms. Prampart S et al. found that a functional decline occurred in 36% of patients and a worse CFS state occurred in 26.8% of patients after 3 months in a similar hospitalized COVID-19 older population [median age: 86 (82–90) years] [42]. We found a close relationship between the onset or persistence of these symptoms and disability progression, while no association was found with radiological sequelae. Regardless of COVID-19, patients aged 65 years or older developed an average of one to two new functional limitations after a hospitalization for sepsis, likely due to a progression of pre-existing chronic conditions, such as HF, chronic kidney disease or chronic respiratory disease, and a residual organ damage [43]. Moreover, protracted bed-resting may accelerate aging-related muscle atrophy processes, pneumonia-induced hypoxia may contribute to accelerating cognitive impairment, and increased pro-inflammatory cytokines could support neurodegenerative processes [44, 45]. Older patients hospitalized for pneumonia are at higher risk of new impairments in ADL and moderate-to-severe cognitive impairment also compared to subjects hospitalized for known disabling conditions, such as myocardial infarction or stroke [46]. As expected, this trend was even more evident in our population with such a high mean age, in which the failure to recover ADL function after hospitalization for medical illnesses is frequent [47]. Therefore, this deterioration is not a problem strictly related to COVID-19. As also confirmed by our findings, pre-existing dependencies and chronic conditions prior to admission, especially cardio-respiratory diseases and the length of hospital stay, play a key role in this process [48]. Among our participants, subjects with higher pre-admission CCI, especially those who suffered from chronic HF and COPD, developed the most significant functional disability. In fact, chronic HF and hospitalization for acute HF, as well as COPD and its exacerbations, are known to be associated with hospital-acquired disability in older patients, resulting in impairment of the ADL [49, 50]. All of these aspects should be taken into account when setting up a tailored geriatric rehabilitation treatment to post-acute COVID-19 to prevent the functional decline trajectory. In our study, all the enrolled patients have been treated with corticosteroids (dexamethasone 6 mg once daily) during hospitalization, according to the evidence and recommendations available at that time [51, 52]. Unfortunately, this did not allow us to investigate the role of this drug class on the sequelae of COVID-19 in our older sample, in which controversial results have been previously found [53]. In our population, no association emerged between the other in-hospital drug treatments administered for COVID-19 and fibrotic-like changes at follow-up. However, we found that a therapeutic dose of anticoagulants, another drug class characterized by mixed results in previous studies [54], was associated with worsening of functional status and frailty condition at follow-up, most likely related at least in part to the presence of atrial fibrillation as comorbidity.

### Study limits

The main strength of our study is the accurate radiological analysis carried out during the follow-up, evaluated together with functional status, frailty, and symptoms in an older population. To the best of our knowledge, no previous studies on such older patients have been performed with such a detailed chest CT assessment, taking into account both qualitative, semi-quantitative, and quantitative characteristics. Another strength is the high mean age of the population, uncommon in the literature [14, 16]. The main limitation of our study is the small sample size, although in line with several other studies on younger populations [14, 16], which did not allow us to perform accurate multivariate analyses of the risk factors. Furthermore, as most of the studies on this topic, the lack of a control group of older patients discharged for other common respiratory diseases does not allow us to perform a direct comparison with other common causes of hospitalization different from COVID-19. After re-evaluating medical records, the baseline HRCT evaluation was performed in an early phase of the acute disease in 12 of 55 patients, likely not allowing to show the maximum extent of SARS-CoV-2 pneumonia in these patients. This could at least partially have affected the comparison between CT scans at the 3-month follow-up. Moreover, our analysis was conducted on survivors, while many of our older patients affected by severe COVID-19 pneumonia died during hospitalization. Pulmonary function tests were performed in a subgroup of patients only (16 patients), given the difficulty of performing spirometry properly in many older comorbid patients. Therefore, it was not possible to perform association analysis with spirometry respiratory parameters. We have no detailed information regarding treatments for post-COVID symptoms, given their non-specificity and the absence of approved targeted therapies. Finally, patients’ impossibility to undergo follow-up evaluations (i.e., important bed-rest syndrome) could have represented a potential selection bias in our cohort.

### Conclusions

Our follow-up study on older patients hospitalized for COVID-19 pneumonia shows that chest CT abnormalities of the acute phase resolved in most cases, although the development of fibrotic-like changes was found in several patients after 6 months, especially males. When present, these fibrotic-like changes appeared to be mild, involving only very limited parts of the lung, and were not likely to affect functional status or frailty condition. Our findings provide additional evidence regarding the progressive trajectory of the manifestations linked to COVID-19 pneumonia and the appearance of “fibrotic-like abnormalities” after 6 months in older patients with mild-to-severe COVID-19. It is not clear which of these abnormalities grouped as “fibrotic-like” are reliably indicative of irreversible disease. On the other hand, the burden of pre-existing comorbidities, such as chronic HF and COPD, and longer hospital stay were risk factors for worsening functional status in our study, likely similar to what has been observed after prolonged hospitalization for any other acute condition in older subjects.

## Supplementary Information

Below is the link to the electronic supplementary material.

Supplementary file1 (DOCX 60 KB)

## Data Availability

The data that support the findings of this study are available from the corresponding author (FS) upon reasonable request.
