# Supplementary material for: Radiological lung sequelae, functional status and symptoms in older patients 3 and 6 months after hospitalization for COVID-19 pneumonia
Source: Intern Emerg Med. 2023 Apr 6;18(4):1075–85. doi: 10.1007/s11739-023-03259-y (PMC10078021; doi:10.1007/s11739-023-03259-y)
Supplement: Supplementary file 1 — Supplementary file1 (DOCX 60 KB) [file 11739_2023_3259_MOESM1_ESM.docx]

Supplementary Material

Internal and Emergency Medicine

**RADIOLOGICAL** **LUNG SEQUELAE, FUNCTIONAL STATUS and SYMPTOMS IN OLDER PATIENTS THREE AND SIX MONTHS AFTER HOSPITALIZATION FOR COVID-19 PNEUMONIA**

Chiara Di Pentima MD* ^1,2^, Sara Cecchini MD* ^3^, Francesco Spannella MD ^1,2^, Federico Giulietti MD, PhD ^1,2^, Massimiliano Allevi MD ^1,2^, Paola Schiavi MD ^1,2^, Francesca Carnevali MD ^3^, Lorenzo Zoppi MD ^3^, Maria Carmela Ciociola MD ^3^, Fiammetta Ventura ^3^, Gina Dragano ^1^, Piero Giordano MD ^1^, Enrico Paci MD ^3^, Riccardo Sarzani MD, PhD ^1,2^

^1^ Internal Medicine and Geriatrics, IRCCS INRCA, Via della Montagnola 81, Ancona, Italy

^2^ Department of Clinical and Molecular Sciences, University “Politecnica delle Marche”, Via Tronto 10/a, Ancona, Italy

^3^ Department of Radiology, IRCCS INRCA, Via della Montagnola 81, Ancona, Italy.

*Chiara Di Pentima and Sara Cecchini contributed equally to the work and should be considered both first authors.

**Corresponding Author:**

Francesco Spannella MD. Internal Medicine and Geriatrics, Department of Clinical and Molecular Sciences, University “Politecnica delle Marche”, IRCCS INRCA, via della Montagnola n. 81, 60127, Ancona, ITALY. Fax +39-071-889232; Phone +39-071-5963241; e-mail: f.spannella@univpm.it

Supplemental Table S1. General baseline characteristics of the study population according to clinical severity (based on the respiratory support needed during hospitalization).

| **General baseline characteristics** | **Oxygen therapy**  **(N° 30)** | **HFNC/CPAP (N° 12)** | **NIV**  **(N° 13)** | **p** |
| --- | --- | --- | --- | --- |
| Age (years) | 82.2 ± 7.3 | 80.1 ± 9.5 | 84.5 ± 3.1 | 0.319 |
| Sex (Male) | 53.3% | 41.7% | 76.9% | 0.244 |
| Overweight/Obesity (BMI >25 Kg/mq) | 53.6% | 58.3% | 75.0% | 0.228 |
| Active/Former smoker | 26.7% | 25.0% | 30.8% | 0.818 |
| CCI | 6 (4-7) | 5 (4-6) | 5 (5-7) | 0.241 |
| GIC classes I-II | 86.7% | 100.0% | 92.3% | 0.414 |
| GIC classes III-IV | 13.3% | 0.0% | 7.7% |  |
| CFS | 3.8 ± 1.2 | 3.3 ± 1.2 | 3.5 ± 1.3 | 0.609 |
| ADL 5-6 | 70.0% | 83.3% | 92.3% | 0.084 |
| ADL 3-4 | 26.7% | 16.7% | 7.7% |  |
| ADL 1-2 | 3.3% | 0.0% | 0.0% |  |
| **Main comorbidities** |  |  |  |  |
| Arterial hypertension | 73.3% | 75.0% | 92.3% | 0.199 |
| Dyslipidemia | 43.3% | 50.0% | 46.2% | 0.814 |
| Anemia (Hb<12 g/dl) | 33.3% | 41.7% | 46.2% | 0.408 |
| Type 2 diabetes mellitus | 30.0% | 33.3% | 53.8% | 0.161 |
| Ischemic heart disease | 26.7% | 25.0% | 15.4% | 0.452 |
| Peripheral artery disease | 20.0% | 8.0% | 23.1% | 0.970 |
| History of heart failure | 20.0% | 8.3% | 15.4% | 0.595 |
| Cognitive impairment | 20.0% | 8.3% | 15.4% | 0.595 |
| COPD | 10.0% | 8.3% | 30.8% | 0.112 |
| Atrial fibrillation | 16.7% | 16.7% | 7.7% | 0.485 |
| Previous Stroke/TIA | 6.7% | 16.7% | 7.7% | 0.760 |
| **Admission laboratory parameters** |  |  |  |  |
| Hb (g/dl) | 12.5 ± 1.7 | 13.3 ± 1.7 | 12.6 1.8 | 0.391 |
| WBC (n/microl) | 6797 ± 3387 | 7512 ± 3388 | 8225 ± 3182 | 0.428 |
| PLT (n/microl) | 209100 ± 75361 | 220000 ± 112584 | 220231 ± 98952 | 0.903 |
| Neutrophils (n/microl) | 4910 ± 2767 | 6025 ± 3208 | 6578 ± 3491 | 0.222 |
| Lymphocytes (n/microl) | 685 (610-955) | 760 (620-1050) | 650 (442-912) | 0.780 |
| eGFR (ml/min/1.73m²) | 67.1 ± 29.1 | 82.4 ± 27.0 | 63.8 ± 24.9 | 0.194 |
| Ferritin (mcg/l) | 478 (269-978) | 417 (233-1146) | 879 (585-1127) | 0.486 |
| CRP (mg/dl)* | 8.3 ± 5.3 | 10.5 ± 5.9 | 13.1 ± 4.3 | 0.028 |
| Hs-troponin T (pg/ml) | 27.9 (14.7-36.8) | 12.9 (8.9-21.1) | 21.2 (15.8-41.7) | 0.012 |
| NT-proBNP (pg/ml) | 847 (394-2612) | 587 (348-1610) | 1156 (454-4005) | 0.316 |
| D-dimer (mcg/l) | 1355 (622-7145) | 970 (640-1380) | 1445 (940-1652) | 0.140 |
| **Admission arterial blood gas parameters** | |  |  |  |
| pH | 7.47 ± 0.47 | 7.48 ± 0.44 | 7.47 ± 0.40 | 0.735 |
| pO2 (mmHg) | 64.5 ± 11.1 | 62.8 ± 11.5 | 69.7 ± 14.8 | 0.314 |
| pCO2 (mmHg) | 33.6 ± 4.4 | 33.7 ± 4.3 | 35.2 ± 5.2 | 0.598 |
| HCO3- (mmol/l) | 24.9 ± 3.6 | 25.9 ± 3.9 | 25.4 ± 5.4 | 0.823 |
| sO2 (%) | 94.0 ± 4.1 | 92.9 ± 4.1 | 96.1 ± 2.2 | 0.119 |
| Lactate (mmol/l) | 1.1 (0.7-1.3) | 1.1 (0.9-1.9) | 1.0 (0.9-1.5) | 0.528 |
| P/F (mmHg) | 276 ± 73 | 243 ± 82 | 250 ± 112 | 0.457 |
| Worst P/F (mmHg) during hospitalization | 213 ± 60 | 127 ± 49 | 140 ± 38 | <0.001 |
| **In-hospital treatment for COVID-19** | | | | |
| Azithromycin | 50.0% | 58.3% | 100.0% | 0.007 |
| Remdesivir | 66.7% | 33.3% | 46.2% | 0.115 |
| Corticosteroids | 100.0% | 100.0% | 100.0% | NA |
| Anticoagulants at prophylactic dose | 46.7% | 75.0% | 46.2% | 0.217 |
| Anticoagulants at therapeutic dose | 50.0% | 25.0% | 53.8% | 0.267 |

* highest value during hospitalization. HFNC/CPAP: High Flow Nasal Cannula / Continuous Positive Airway Pressure; NIV: Non-Invasive Ventilation; CCI: Charlson Comorbidity Index; GIC: Geriatric Index of Comorbidity; CFS: Clinical Frailty Scale; ADL: Activities of Daily Living; Hb: Hemoglobin; COPD: chronic obstructive pulmonary disease; TIA: transient ischemic attack; WBC: white blood cells count; PLT: platelets count; eGFR: estimated glomerular filtration rate; CRP: C-reactive protein; NT-proBNP: N-terminal pro-B-type natriuretic peptide; P/F: ratio of arterial oxygen partial pressure (PaO2 in mmHg) to fractional inspired oxygen (FiO2 expressed as a fraction, not a percentage); NA: not applicable.

Supplemental Table S2. General baseline characteristics of the study population according to CT severity score on admission.

| **General baseline characteristics** | **Mild (N° 18)** | **Moderate (N° 30)** | **Severe (N° 7)** | **p** |
| --- | --- | --- | --- | --- |
| Age (years) | 84.8 ± 3.8 | 80.0 ± 7.5 | 85.0 ± 9.6 | 0.043 |
| Sex (Male) | 33.3% | 70.0% | 57.1% | 0.079 |
| Overweight/Obesity (BMI >25 Kg/mq) | 37.5% | 69.0% | 71.4% | 0.057 |
| Active/Former smoker | 27.8% | 23.3% | 42.9% | 0.641 |
| CCI | 6.5 (5-8) | 5 (4-7) | 6 (5-7) | 0.024 |
| GIC classes I-II | 88.9% | 93.3% | 85.7% | 0.767 |
| GIC classes III-IV | 11.1% | 6.7% | 14.3% |  |
| CFS | 4.2 ± 1.3 | 3.0 ± 0.9 | 4.7 ± 1.2 | <0.001 |
| ADL 5-6 | 72.2% | 86.7% | 57.1% | 0.859 |
| ADL 3-4 | 22.2% | 13.3% | 42.9% |  |
| ADL 1-2 | 5.6% | 0.0% | 0.0% |  |
| **Main comorbidities** |  |  |  |  |
| Arterial hypertension | 66.7% | 83.0% | 85.7% | 0.191 |
| Dyslipidemia | 38.9% | 50.0% | 42.9% | 0.677 |
| Anemia (Hb<12 g/dl) | 61.1% | 23.3% | 14.3% | 0.105 |
| Type 2 diabetes mellitus | 33.3% | 36.7% | 42.9% | 0.666 |
| Ischemic heart disease | 27.8% | 23.3% | 7.7% | 0.494 |
| Peripheral artery disease | 22.2% | 17.0% | 14.3% | 0.591 |
| History of heart failure | 22.2% | 13.3% | 14.3% | 0.501 |
| Cognitive impairment | 33.3% | 0.0% | 42.9% | 0.501 |
| COPD | 16.7% | 6.7% | 42.9% | 0.346 |
| Atrial fibrillation | 27.8% | 10.0% | 0.0% | 0.121 |
| Previous Stroke/TIA | 17.0% | 7.0% | 0.0% | 0.149 |
| **Admission laboratory parameters** |  |  |  |  |
| Hb (g/dl) | 12.0 ± 1.5 | 13.0 ± 1.8 | 12.4 ± 1.8 | 0.114 |
| WBC (n/microl) | 5996 ± 1432 | 7907 ± 3173 | 7980 ± 3223 | 0.132 |
| PLT (n/microl) | 195611 ± 79377 | 212700 ± 85291 | 267714 ± 114654 | 0.188 |
| Neutrophils (n/microl) | 4364 ± 3454 | 5973 ± 2644 | 6764 ± 3253 | 0.133 |
| Lymphocytes (n/microl) | 645 (527-1000) | 770 (630-883) | 860 (430-1030) | 0.469 |
| eGFR (ml/min/1.73m²) | 60.9 ± 18.7 | 69.2 ± 30.1 | 93.8 ± 28.1 | 0.027 |
| Ferritin (mcg/l) | 352 (227-626) | 929 (422-1136) | 551 (233-1095) | 0.006 |
| CRP (mg/dl)* | 7.0 ± 4.7 | 10.5 ± 5.1 | 15.1 ± 5.2 | 0.002 |
| Hs-troponin T (pg/ml) | 28.2 (13.3-36.3) | 18.9 (11.5-32.8) | 19.6 (15.2-33.8) | 0.294 |
| NT-proBNP (pg/ml) | 892 (414-2501) | 576 (351-2187) | 1279 (890-3132) | 0.456 |
| D-dimer (mcg/l) | 1120 (513-2368) | 1245 (670-3668) | 1370 (920-2510) | 0.463 |
| **Admission arterial blood gas parameters** | |  |  |  |
| pH | 7.47 ± 0.04 | 7.47 ± 0.04 | 7.51 ± 0.04 | 0.034 |
| pO2 (mmHg) | 68.7 ± 13.2 | 63.6 ± 9.5 | 64.4 ± 18.9 | 0.384 |
| pCO2 (mmHg) | 34.0 ± 5.8 | 33.1 ± 3.7 | 38.0 ± 5.8 | 0.035 |
| HCO3- (mmol/l) | 24.4 ± 3.1 | 24.5 ± 3.6 | 31 ± 4.3 | 0.002 |
| sO2 (%) | 95.1 ± 2.9 | 94.3 ± 2.9 | 92.9 ± 7.2 | 0.426 |
| Lactate (mmol/l) | 1.1 (0.7-2.0) | 1.1 (0.7-1.2) | 1.1 (1.0-1.4) | 0.440 |
| P/F (mmHg) | 289 ± 100 | 259 ± 65 | 213 ± 110 | 0.127 |
| Worst P/F (mmHg) during hospitalization | 190 ± 67 | 178 ± 67 | 142 ± 55 | 0.271 |
| **Respiratory support needed during hospitalization** | | | | |
| Oxygen therapy | 66.7% | 11.1% | 22.2% | 0.161 |
| HFNC/CPAP | 53.3% | 26.7% | 20.0% |  |
| NIV | 28.6% | 28.6% | 42.9% |  |
| **In-hospital treatment for COVID-19** | | | | |
| Azithromycin | 66.7% | 56.7% | 85.7% | 0.666 |
| Remdesivir | 72.2% | 50.0% | 28.6% | 0.037 |
| Corticosteroids | 100.0% | 100.0% | 100.0% | NA |
| Anticoagulants at prophylactic dose | 27.8% | 63.3% | 71.4% | 0.016 |
| Anticoagulants at therapeutic dose | 66.7% | 36.7% | 28.6% | 0.037 |

* highest value during hospitalization. CCI: Charlson Comorbidity Index; GIC: Geriatric Index of Comorbidity; CFS: Clinical Frailty Scale ADL: Activities of Daily Living; Hb: Hemoglobin; COPD: chronic obstructive pulmonary disease; TIA: transient ischemic attack; WBC: white blood cells count; PLT: platelets count; eGFR: estimated glomerular filtration rate; CRP: C-reactive protein; NT-proBNP: N-terminal pro-B-type natriuretic peptide; P/F: ratio of arterial oxygen partial pressure (PaO2 in mmHg) to fractional inspired oxygen (FiO2 expressed as a fraction, not a percentage); HFNC/CPAP: High Flow Nasal Cannula/ Continuous Positive Airway Pressure; NIV: Non Invasive Ventilation; NA: not applicable.

Supplemental Table S3. General baseline characteristics of the study population according to the presence of fibrotic-like changes at 6-month follow-up chest HRCT.

| **General baseline characteristics** | **No fibrotic-like changes**  **(N° 33)** | | | **Fibrotic-like changes**  **(N° 22)** | | **p** |
| --- | --- | --- | --- | --- | --- | --- |
| Age (years) | 81.4 ± 7.1 | | | 83.6 ± 7.2 | | 0.273 |
| Sex (Male) | 45.5% | | | 72.7% | | 0.046 |
| Overweight/Obesity (BMI >25 Kg/mq) | 60.0% | | | 59.1% | | 0.947 |
| Active/Former smoker | 21.2% | | | 36.4% | | 0.216 |
| CCI | 6 (4-7) | | | 5 (4-7) | | 0.937 |
| GIC classes I-II | 90.9% | | | 90.9% | | 1.000 |
| GIC classes III-IV | 9.1% | | | 9.1% | |  |
| CFS | 3.5 ± 1.2 | | | 3.8 ± 1.4 | | 0.356 |
| ADL 5-6 | 81.8% | | | 72.7% | | 0.639 |
| ADL 3-4 | 15.2% | | | 27.3% | |  |
| ADL 1-2 | 3.0% | | | 0.0% | |  |
| **Main comorbidities** |  | | |  | |  |
| Arterial hypertension | 69.7% | | | 90.9% | | 0.096 |
| Dyslipidemia | 48.5% | | | 40.9% | | 0.580 |
| Anemia (Hb<12 g/dl) | 42.4% | | | 31.8% | | 0.428 |
| Type 2 diabetes mellitus | 36.4% | | | 36.4% | | 1.000 |
| Ischemic heart disease | 21.2% | | | 27.3% | | 0.604 |
| Peripheral artery disease | 15.2% | | | 22.7% | | 0.498 |
| History of heart failure | 21.2% | | | 9.1% | | 0.289 |
| Cognitive impairment | 12.1% | | | 22.7% | | 0.459 |
| COPD | 15.2% | | | 13.6% | | 0.876 |
| Atrial fibrillation | 15.2% | | | 13.6% | | 0.876 |
| Previous Stroke/TIA | 6.1% | | | 13.6% | | 0.379 |
| **Admission laboratory parameters** |  | | |  | |  |
| Hb (g/dl) | 12.3 ± 1.6 | | | 13.2 ± 1.9 | | 0.063 |
| WBC (n/microl) | 7002 ± 3215 | | | 7724 ± 3531 | | 0.436 |
| PLT (n/microl) | 204363 ± 77854 | | | 228727 ± 102698 | | 0.322 |
| Neutrophils (n/microl) | 5092 ± 2993 | | | 6230 ± 3134 | | 0.181 |
| Lymphocytes (n/microl) | 700 (590-1010) | | | 705 (528-860) | | 0.323 |
| eGFR (ml/min/1.73m²) | 68.7 ± 27.6 | | | 71.0 ± 29.2 | | 0.764 |
| Ferritin (mcg/l) | 541 (263-1046) | | | 887 (363-1140) | | 0.149 |
| CRP (mg/dl)* | 10.0 ± 5.4 | | | 9.9 ± 5.7 | | 0.930 |
| Hs-troponin T (pg/ml) | 26.7 (14.4-36.3) | | | 18.3 (10.9-29.1) | | 0.148 |
| NT-proBNP (pg/ml) | 602 (394-2363) | | | 969 (374-1843) | | 0.725 |
| D-dimer (mcg/l) | 1360 (920-3410) | | | 1110 (595-2085) | | 0.352 |
| **Admission arterial blood gas parameters** | | | |  | |  |
| pH | 7.48 ± 0.04 | | | 7.47 ± 0.04 | | 0.521 |
| pO2 (mmHg) | 66.0 ± 11.1 | | | 64.6 ± 13.9 | | 0.671 |
| pCO2 (mmHg) | 33.8 ± 5.3 | | | 34.4 ± 3.3 | | 0.651 |
| HCO3- (mmol/l) | 25.3 ± 3.9 | | | 25.2 ± 4.5 | | 0.945 |
| sO2 (%) | 94.8 ± 3.0 | | | 93.6 ± 4.8 | | 0.296 |
| Lactate (mmol/l) | 1.1 (0.9-1.5) | | | 1.0 (0.7-1.3) | | 0.337 |
| P/F (mmHg) | 255 ± 82 | | | 274 ± 91 | | 0.402 |
| Worst P/F (mmHg) during hospitalization | 183 ± 75 | | | 168 ± 50 | | 0.364 |
| **Respiratory support needed during hospitalization** | | | | | | |
| Oxygen therapy | 57.6% | | | 50.0% | | 0.792 |
| HFNC/CPAP | 18.2% | | | 27.3% | |  |
| NIV | 24.2% | | | 22.7% | |  |
| **In-hospital treatment for COVID-19** | | | | | | |
| Azithromycin | | 63.6% | 63.6% | | 1.000 | |
| Remdesivir | | 57.6% | 50.0% | | 0.580 | |
| Corticosteroids | | 100.0% | 100.0% | | NA | |
| Anticoagulants at prophylactic dose | | 54.5% | 50.0% | | 0.741 | |
| Anticoagulants at therapeutic dose | | 45.5% | 45.5% | | 1.000 | |

* highest value during hospitalization. CCI: Charlson Comorbidity Index; GIC: Geriatric Index of Comorbidity; CFS: Clinical Frailty Scale ADL: Activities of Daily Living; Hb: Hemoglobin; COPD: chronic obstructive pulmonary disease; TIA: transient ischemic attack; WBC: white blood cells count; PLT: platelets count; eGFR: estimated glomerular filtration rate; CRP: C-reactive protein; NT-proBNP: N-terminal pro-B-type natriuretic peptide; P/F: ratio of arterial oxygen partial pressure (PaO2 in mmHg) to fractional inspired oxygen (FiO2 expressed as a fraction, not a percentage); HFNC/CPAP: High Flow Nasal Cannula/ Continuous Positive Airway Pressure; NIV: Non Invasive Ventilation; NA: not applicable.

Supplemental Table S4. General baseline characteristics of the study population according to trends of functional status and frailty condition

| **General baseline characteristics** | **No reduction in ADL (N° 49)** | **Reduction in ADL**  **(N° 6)** | **p** | **No increase in CFS (N° 30)** | **Increase in CFS (N° 25)** | **p** |
| --- | --- | --- | --- | --- | --- | --- |
| Age (years) | 82.0 ± 4.5 | 84.2 ± 2.9 | 0.484 | 82.2 ± 7.6 | 82.3 ± 6.7 | 0.986 |
| Sex (Male) | 57.1% | 50.0% | 0.739 | 63.3% | 48.0% | 0.286 |
| Overweight/Obesity (BMI >25 Kg/mq) | 61.7% | 40.0% | 0.380 | 69.0% | 47.8% | 0.160 |
| Active/Former smoker | 20.4% | 83.3% | 0.001 | 20.0% | 36.0% | 0.231 |
| CCI | 5 (4-7) | 7 (6-10) | 0.020 | 5 (4-7) | 6 (4-7) | 0.492 |
| GIC classes I-II | 91.8% | 83.3% |  | 90.0% | 92.0% | 0.797 |
| GIC classes III-IV | 8.2% | 16.7% | 0.450 | 10.0% | 8.0% |  |
| CFS | 3.4 ± 1.2 | 5.2 ± 1.0 | 0.001 | 3.5 ± 1.3 | 3.8 ± 1.3 | 0.348 |
| ADL 5-6 | 79.6% | 66.7% | 0.590 | 76.7% | 80.0% | 0.958 |
| ADL 3-4 | 18.4% | 33.3% |  | 23.3% | 16.0% |  |
| ADL 1-2 | 2.0% | 0.0% |  | 0.0% | 4.0% |  |
| **Main comorbidities** |  |  |  |  |  |  |
| Arterial hypertension | 81.6% | 50.0% | 0.110 | 80.0% | 76.0% | 0.721 |
| Dyslipidemia | 46.9% | 33.3% | 0.680 | 53.3% | 36.0% | 0.278 |
| Anemia (Hb<12 g/dl) | 36.7% | 50.0% | 0.660 | 36.7% | 40.0% | 0.800 |
| Type 2 diabetes mellitus | 38.8% | 16.7% | 0.400 | 53.3% | 16.0% | 0.005 |
| Ischemic heart disease | 20.4% | 50.0% | 0.140 | 20.0% | 28.0% | 0.537 |
| Peripheral artery disease | 16.3% | 33.3% | 0.300 | 13.3% | 24.0% | 0.484 |
| History of heart failure | 12.2% | 50.0% | 0.050 | 16.7% | 16.0% | 1.00 |
| Cognitive impairment | 14.3% | 33.3% | 0.250 | 13.3% | 20.0% | 0.716 |
| COPD | 8.2% | 66.7% | 0.003 | 6.7% | 24.0% | 0.123 |
| Atrial fibrillation | 12.2% | 33.3% | 0.210 | 6.7% | 24.0% | 0.123 |
| Previous Stroke/TIA | 10.2% | 0.0% | 1.000 | 10.0% | 8.0% | 1.000 |
| **Admission laboratory parameters** |  |  |  |  |  |  |
| Hb (g/dl) | 12.7 ± 1.7 | 12.0 ± 0.7 | 0.329 | 12.4 ± 1.7 | 12.9 ± 1.7 | 0.324 |
| WBC (n/microl) | 7499 ± 3410 | 5587 ± 2082 | 0.187 | 6962 ± 2822 | 7685 ± 3882 | 0.428 |
| PLT (n/microl) | 216204 ± 90505 | 197000 ± 75011 | 0.621 | 214233 ± 77448 | 213960 ± 101925 | 0.991 |
| Neutrophils (n/microl) | 5760 ± 3140 | 3813 ± 1816 | 0.145 | 5023 ± 2180 | 6176 ± 3841 | 0.191 |
| Lymphocytes (n/microl) | 750 (550-935) | 630 (510-1518) | 0.770 | 730 (592-922) | 670 (540-1040) | 0.886 |
| eGFR (ml/min/1.73m²) | 70.7 ± 27.4 | 61.8 ± 34.0 | 0.421 | 65.1 ± 29.3 | 75.0 ± 26.0 | 0.197 |
| Ferritin (mcg/l) | 639 (315-1123) | 314 (226-982) | 0.200 | 644 (361-1057) | 541 (279-1127) | 0.673 |
| CRP (mg/dl)* | 10.2 ± 5.6 | 7.5 ± 3.9 | 0.248 | 10.9 ± 6.2 | 8.8 ± 4.5 | 0.165 |
| Hs-troponin T (pg/ml) | 19.6 (12.6-35.1) | 27.3 (18.7-36.4) | 0.680 | 28.0 (17.6-46.0) | 19.0 (11.3-30.0) | 0.065 |
| NT-proBNP (pg/ml) | 771 (394-1756) | 1969 (509-6810) | 0.210 | 969 (356-2885) | 703 (417-1647) | 0.710 |
| D-dimer (mcg/l) | 1190 (685-2290) | 2170 (895-15560) | 0.230 | 1350 (915-3655) | 1160 (590-2100) | 0.450 |
| **Admission arterial blood gas parameters** | |  |  |  |  |  |
| pH | 7.47 ± 0.04 | 7.50 ± 0.05 | 0.180 | 7.47 ± 0.05 | 7.49 ± 0.04 | 0.028 |
| pO2 (mmHg) | 63.7 ± 11.1 | 78.8 ± 12.8 | 0.030 | 65.6 ± 11.9 | 65.2 ± 12.7 | 0.909 |
| pCO2 (mmHg) | 34.3 ± 4.6 | 32.0 ± 4.3 | 0.255 | 34.7 ± 5.0 | 33.2 ± 4.0 | 0.229 |
| HCO3- (mmol/l) | 25.2 ± 4.1 | 25.5 ± 4.2 | 0.897 | 25.1 ± 4.9 | 25.5 ± 3.3 | 0.731 |
| sO2 (%) | 94.1 ± 3.8 | 96.3 ± 3.0 | 0.171 | 94.0 ± 4.3 | 94.8 ± 2.9 | 0.463 |
| Lactate (mmol/l) | 1.1 (0.8-1.37) | 1.0 (0.7-1.3) | 0.610 | 1.1 (0.8-1.3) | 1.1 (0.8-1.5) | 0.793 |
| P/F (mmHg) | 252 ± 80 | 355 ± 80 | 0.004 | 269 ± 79 | 256 ± 94 | 0.578 |
| Worst P/F (mmHg) during hospitalization | 172 ± 67 | 223 ± 45 | 0.075 | 187 ± 68 | 165 ± 64 | 0.237 |
| **Respiratory support needed during hospitalization** | | | | | | |
| Oxygen therapy | 53.1% | 66.7% | 0.940 | 66.7% | 40.0% | 0.115 |
| HFNC/CPAP | 24.5% | 0.0% |  | 13.3% | 32.0% |  |
| NIV | 22.4% | 33.3% |  | 20.0% | 28.0% |  |
| **In-hospital treatment for COVID-19** | | | | | | |
| Azithromycin | 61.2% | 83.3% | 0.292 | 56.7% | 72.0% | 0.239 |
| Remdesivir | 53.1% | 66.7% | 0.531 | 53.3% | 56.0% | 0.843 |
| Corticosteroids | 100.0% | 100.0% | NA | 100.0% | 100.0% | NA |
| Anticoagulants at prophylactic dose | 59.2% | 0.0% | 0.007 | 70.0% | 32.0% | 0.005 |
| Anticoagulants at therapeutic dose | 40.8% | 83.3% | 0.050 | 30.0% | 64.0% | 0.012 |

* highest value during hospitalization. CCI: Charlson Comorbidity Index; GIC: Geriatric Index of Comorbidity; CFS: Clinical Frailty Scale ADL: Activities of Daily Living; Hb: Hemoglobin; COPD: chronic obstructive pulmonary disease; TIA: transient ischemic attack; WBC: white blood cells count; PLT: platelets count; eGFR: estimated glomerular filtration rate; CRP: C-reactive protein; NT-proBNP: N-terminal pro-B-type natriuretic peptide; P/F: ratio of arterial oxygen partial pressure (PaO2 in mmHg) to fractional inspired oxygen (FiO2 expressed as a fraction, not a percentage); HFNC/CPAP: High Flow Nasal Cannula/ Continuous Positive Airway Pressure; NIV: Non Invasive Ventilation, NA: not applicable.
